# Long noncoding RNA ANRIL as a novel biomarker of lymph node metastasis and prognosis in human cancer: a meta-analysis

**DOI:** 10.18632/oncotarget.21825

**Published:** 2017-10-11

**Authors:** Han Wang, Yang Liu, Jianhua Zhong, Chenglong Wu, Yuantang Zhong, Gang Yang, Jinghua Zhang, Aifa Tang

**Affiliations:** ^1^ Department of Urinary Surgery, Shenzhen Second People's Hospital, The First Affiliated Hospital of Shenzhen University, Shenzhen, Guangdong, China; ^2^ Guangzhou Medical University, Guangzhou, Guangdong, China

**Keywords:** ANRIL, lncRNA, cancers, lymph node metastasis, overall survival

## Abstract

Dysregulation of the long noncoding RNA antisense noncoding RNA in the INK4 locus (ANRIL) has been reported in various solid tumors. We performed a synthetic analysis to clarify the clinical value of ANRIL as a prognostic indicator in malignant tumors. Article collection was conducted using several electronic databases, including PubMed, Web of Science, Medline, OVID and Embase (up to July 14 2017). Thirteen original studies and 1172 total patients were included in the meta-analysis. There was a significant positive association between the high expression level of ANRIL and lymph node metastasis (OR = 4.77, 95% CI: 2.30–9.91, *P* < 0.001) by a random effects model (I^2^ = 73.2, *P* = 0.001) and negative association with poor grade cancer (OR = 3.44, 95% CI: 1.68–7.08) by a random-effects model (I^2^ = 77.9, *P* = 0.000). The results of the meta-analysis showed that overexpression of ANRIL is positively related to poor overall survival (OS) (pooled HR = 2.12, 95% CI: 1.78–2.53, *P* < 0.0001) by a fixed-effects model (I^2^ = 0%, *P* = 0.654) and poor disease-free survival (DFS) (HR = 2.10, 95% CI: 1.51–2.92, *P* < 0.001) by a fixed-effects model (I^2^ = 13.3%, *P* = 0.315) in human solid cancers. Statistically significant associations were also found with cancer type, analysis method, sample size, and follow-up time. In conclusion, ANRIL may serve as a novel biomarker for indicating lymph node metastasis and prognosis in human cancer.

## INTRODUCTION

Owing to its increasing incidence and associated mortality, cancer is becoming the leading cause of death and represents a major public health concern worldwide. It is expected that 1,688,780 new cases of cancer and 600,920 cancer-related deaths will occur in the United States alone in 2017. According to published studies, an estimated 4292,000 new cancer cases and 2814,000 cancer deaths will occur in 2015 in China [1, 2]. Although clinical treatments such as surgery, chemotherapy, radiotherapy, targeted therapy, or other comprehensive treatments can improve the prognosis of patients with cancer [3], the 5-year survival rate remains low owing to the malignant progression of tumors [4] and the lack of effective early diagnostic methods. Therefore, the identification of potential diagnostic and prognostic biomarkers for monitoring patients with cancer to observe disease progression is critical.

Long noncoding RNA (lncRNA) are mRNA-like transcripts with a length of more than 200 nucleotides that lack an open reading frame [5]. Such transcripts were originally considered as genomic “noise” [6]; however, increasing evidence suggests that lncRNAs act as critical regulators in diverse diseases and cellular processes, such as the regulation of gene expression regulation and post-translational processing [7–9]. Recent studies on lncRNAs have been reported in a variety of tumors [10–13]. In particular, several lncRNAs are involved in tumor proliferation, migration, and invasion, of which some are considered as representing promising prognostic markers [14–16].

Antisense noncoding RNA in the *INK4* locus (ANRIL) is transcribed in the antisense orientation of the *INK4B-ARF-INK4A* gene cluster, to generate a 3834 nucleotide RNA transcript that includes 19 exons [17]. ANRIL was first detected from patients with familial melanoma by Pasmant et al. in 2007 [18]. Subsequently, numerous studies have reported that ANRIL is independently associated with several other forms of cancer at the genome-wide level and is upregulated in many cancers including colorectal cancer [19], nasopharyngeal carcinoma [20], gallbladder cancer [21], hepatocellular carcinoma [22], non-small cell lung cancer [23], cervical cancer [24], gastric cancer [25], serous ovarian cancer [26], and thyroid cancer [27]. In addition, high expression of ANRIL was significantly related to clinicopathological parameters such as lymph node metastasis (LNM) and overall survival (OS) [19–31]. However, as such individual studies provided discrete consequences and were limited by sample size, no consensus has yet been reached regarding the prognostic value of ANRIL in patients with cancer. Therefore, this meta-analysis was conducted to elucidate the prognostic value of ANRIL as a novel candidate biomarker in malignant tumors.

## RESULTS

### Characteristics of eligible studies

**Figure 1:**
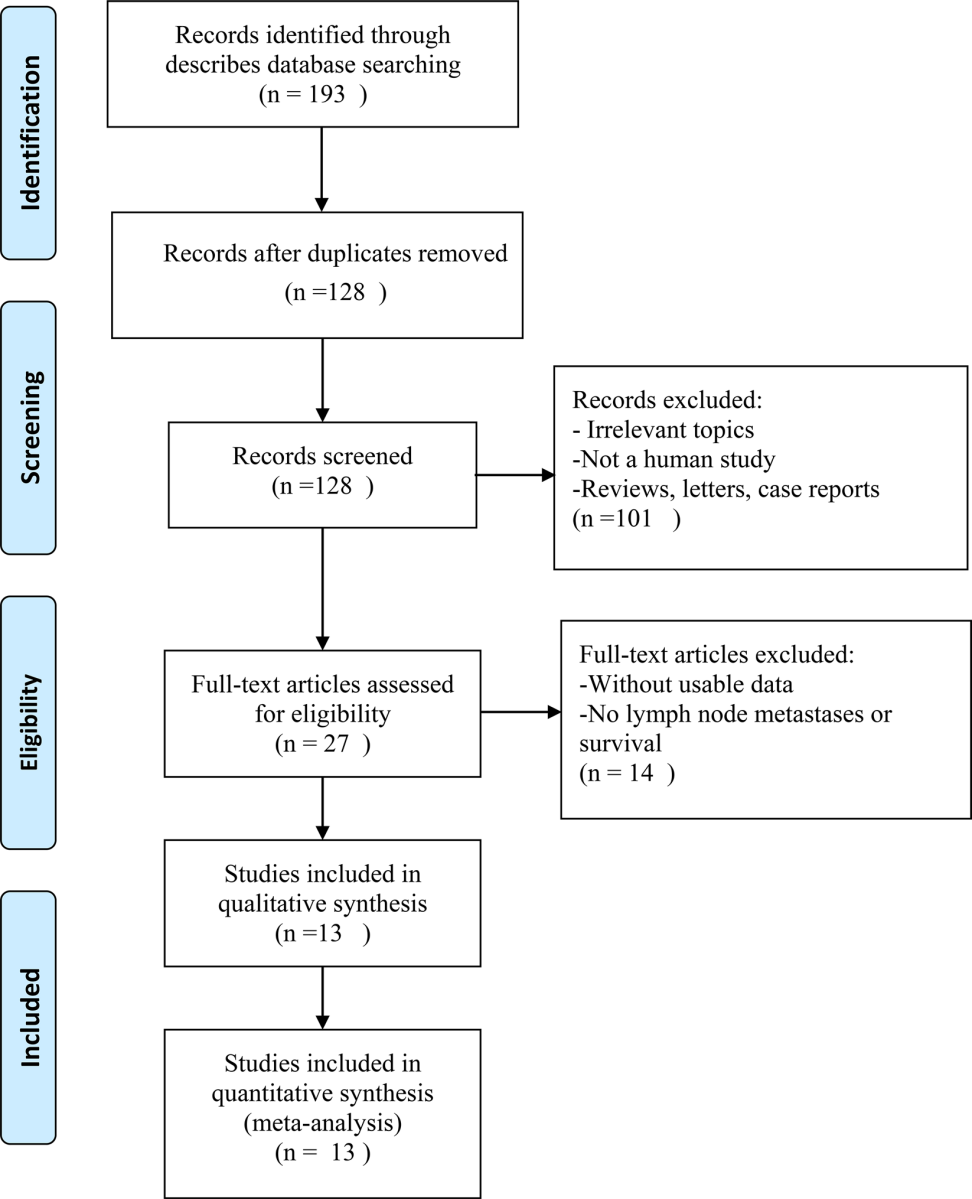
The flow diagram of this meta-analysis

As displayed in the flow diagram (Figure 1), a total of 193 articles were identified in our initial screening from which 65 duplicate reports were excluded. After screening the titles and abstracts carefully, irrelevant studies and those not in humans were excluded, with 27 potentially eligible articles selected. Then, after further assessment of the full studies, 14 articles were excluded owing to lack of usable data or information regarding LNM or survival outcomes. Finally, a total of 13 articles met the selection criteria [19–31].

The studies, which were published between 2014 and 2017, contained a total of 1172 patients with a mean patients sample size of 90.15 (range 53–120). Among the 13 studies, two focused on non–small cell lung cancer (NSCLC), two on gastric cancer (GC), one on hepatocellular carcinoma (HCC), one on serous ovarian cancer (SOC), one on gallbladder cancer (GBC), one on epithelial ovarian cancer (EOC), two on colorectal cancer (CC), one on thyroid cancer (TC), one on nasopharyngeal carcinoma (NPC), and one on cervical cancer (CEC). The expression of ANRIL was detected by quantitative reverse transcription polymerase chain reaction (qRT-PCR) and standardized to *GAPDH* or β-actin. All the patients in the retrieved articles were divided into two groups based on the expression of ANRIL (high or low). The main features of the eligible studies are integrated in (Table 1). The Newcastle-Ottawa Scale (NOS) confirmed that all studies were of good quality, as shown in (Table 2).

**Table 1:** Characteristics of studies in this meta-analysis

Study	year	country	Cancer type	Total number	Tumor stage	Detection method	Cut-off	ANRIL expression				Survival analysis	Multivariate analysis	HR statistic	Hazard ratios (95% CI)	Follow-up period
								High expression	High with LNM	Low expression	Low with LNM					
Nie	2014	China	NSCLC	68	I-III	RT-qPCR	Median	34	23	34	15	OS/DFS	NO	Rep	2.754 [1.142, 6.644]	< 5
Zhang	2014	China	GC	120	I-IV	RT-qPCR	Median	55	34	65	37	OS/DFS	YES	Rep	1.743 [1.036, 2.933]	≥ 5
Hua	2015	China	HCC	92	I-IV	RT-qPCR	NR	46	NR	46	NR	OS	YES	Rep	2.684 [1.534, 6.992]	< 5
Lin	2015	China	NSCLC	87	I-III	RT-qPCR	NR	48	40	39	7	OS	YES	Rep	2.538 [1.374, 5.452]	≥ 5
Qiu	2015	China	SOC	68	I-IV	RT-qPCR	Median	34	27	34	13	OS	YES	Rep	1.895 [1.018, 3.530]	≥ 5
Deng	2016	China	GC	100	NR	RT-qPCR	Mean	57	NR	43	NR	OS	NO	SC	1.580 [1.090, 2.290]	≥ 5
Liu	2016	China	GBC	84	I-IV	RT-qPCR	Median	42	NR	42	NR	OS	NO	SC	1.280 [0.400, 4.140]	≥ 5
Qiu	2016	China	EOC	102	I-IV	RT-qPCR	Median	51	NR	51	NR	OS	YES	Rep	1.928 [1.118, 3.324]	≥ 5
Sun	2016	China	CC	97	I-IV	RT-qPCR	Mean	53	NR	44	NR	OS	NO	SC	2.880 [1.770, 4.700]	≥ 5
Sun	2016	China	CC	108	I-IV	RT-qPCR	Median	82	53	26	5	OS	NO	SC	2.630 [1.400, 4.950]	≥ 5
Zhao	2016	China	TC	105	I-IV	RT-qPCR	NR	53	39	52	19	NR	NR	NR	NR	NR
Zou	2016	China	NPC	88	I-IV	RT-qPCR	Median	44	NR	44	NR	OS/DFS	YES	Rep	4.340 [2.691, 27.268]	≥ 5
Zhang	2017	China	CEC	53	I-III	RT-qPCR	Median	27	10	26	2	OS	YES	Rep	2.715 [1.386, 7.364]	≥ 5

NSCLC: non–small cell lung cancer; GC: gastric cancer; HCC: hepatocellular carcinoma; SOC: serous ovarian cancer; GBC: gallbladder cancer; EOC: epithelial ovarian cancer; CC: colorectal cancer; TC: thyroid cancer; NPC: nasopharyngeal carcinoma; CEC: cervical cancer.

**Table 2:** Study quality assessment according to the Newcastle-Ottawa scale

Author	Country	Adequacy of case definition	Representativeness of the cases	Select of controls	Definition of controls	Comparability case/controls	Ascertainment of exposure	Same method of ascertainment	Nonreponse rate
Nie	China	★	★	★	NA	★★	★	★	NA
Zhang	China	★	★	★	NA	★★	★	★	NA
Hua	China	★	★	★	NA	★★	★	★	NA
Lin	China	★	★	★	NA	★★	★	★	NA
Qiu	China	★	★	★	NA	★★	★	★	NA
Deng	China	★	★	★	NA	★★	★	★	NA
Liu	China	★	★	★	NA	★★	★	★	NA
Qiu	China	★	★	★	NA	★★	★	★	NA
Sun	China	★	★	★	NA	★★	★	★	NA
Sun	China	★	★	★	NA	★★	★	★	NA
Zhao	China	★	★	★	NA	★★	★	★	NA
Zou	China	★	★	★	NA	★★	★	★	NA
Zhang	China	★	★	★	NA	★★	★	★	NA

Note: A study can be awarded a maximum of one star for each numbered item within the Selection and Exposure categories. A maximum of two stars can be given for Comparability.

### Meta-analysis results

#### Association between lncRNA ANRIL and clinicopathological features

**Figure 2:**
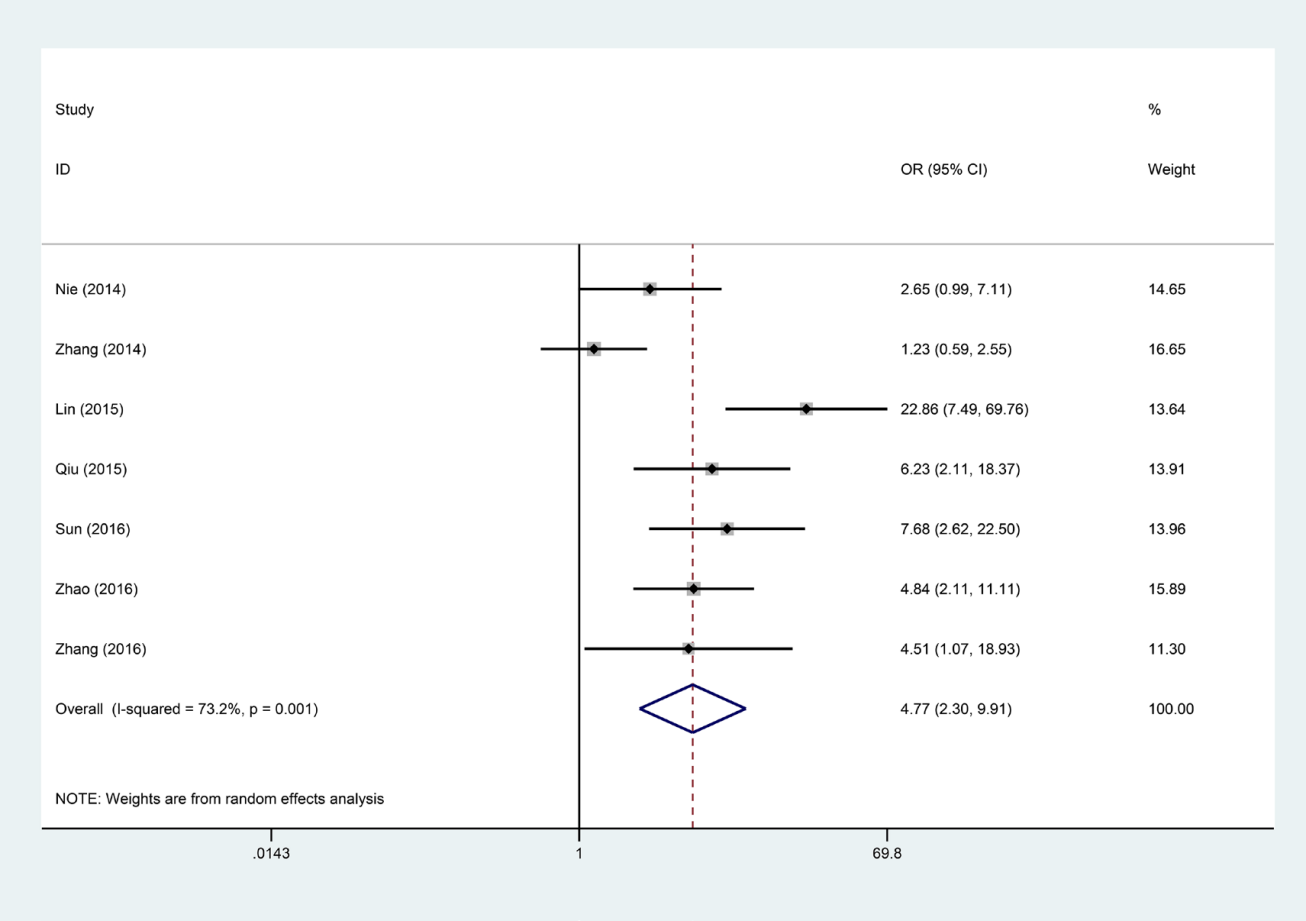
Forest plot for the association between ANRIL expression levels with LNM

Data from 7 articles encompassing a total of 609 patients were selected for analysis of the association between the lncRNA ANRIL and LNM. The random-effects model was adopted owing to significant heterogeneity (I^2^ = 73.2, *P* = 0.001). The results showed that the OR was 4.77 with 95% CI: 2.30–9.91 (*P* < 0.001) (Figure 2). Sensitivity analysis was performed to determine the source of heterogeneity of the statistical results. The analysis showed that the heterogeneity was significantly diminished after the Zhang 2014 study was excluded (I^2^ = 43, *P* = 0.119) without affecting the results (OR = 6.03, 95% CI: 3.94–9.25, *P* < 0.0001) (Supplementary Figure 1). Moreover, the results showed a significant difference in the incidence of LNM between the two groups. Taken together, the analysis suggests that high ANRIL expression indicates a higher propensity to develop LNM.

An association between high expression of ANRIL and advanced clinical stage was also found (OR = 3.44, 95% CI: 1.68–7.08) through application of the random-effects model (I^2^ = 77.9, *P* = 0.000). The analysis showed that the heterogeneity was significantly diminished after the Sun 2016 study was excluded (I^2^ = 0%, *P* = 0.574) also without affecting the results (OR = 4.61, 95% CI: 3.22–6.61, *P* < 0.0001). There was no significant association between high expression level of ANRIL and histologic grade (OR = 1.42, 95% CI: 0.32–6.27, *P* = 0.646) or tumor size (OR = 1.77, 95% CI: 0.63–4.93, *P* = 0.278) (Table 3).

**Table 3:** Meta-analysis results of the associations of high lncRNA ANRIL expression level with clinicopathological features

Clinicopathological parameters	Studies (*n*)	Number of patients	OR (95% CI)	*p*-value	Heterogeneity		
					I^2^ (%)	*P*_Q_	Model
Lymph node metastasis (Yes vs. No)	7	609	4.77 [2.30, 9.91]	< 0.001	73.2	0.001	Random effects
TNM stage (III-IV vs. I-II)	8	751	3.44 [1.68, 7.08]	0.001	77.9	0.000	Random effects
Histologic grade (High vs. Low)	3	262	1.42 [0.32, 6.27]	0.646	86.7	0.001	Random effects
Tumor size (≥ 5 cm vs. < 5 cm)	4	388	1.77 [0.63, 4.93]	0.278	81.7	0.001	Random effects

### Association between lncRNA ANRIL and disease-free survival (DFS)

**Figure 3:**
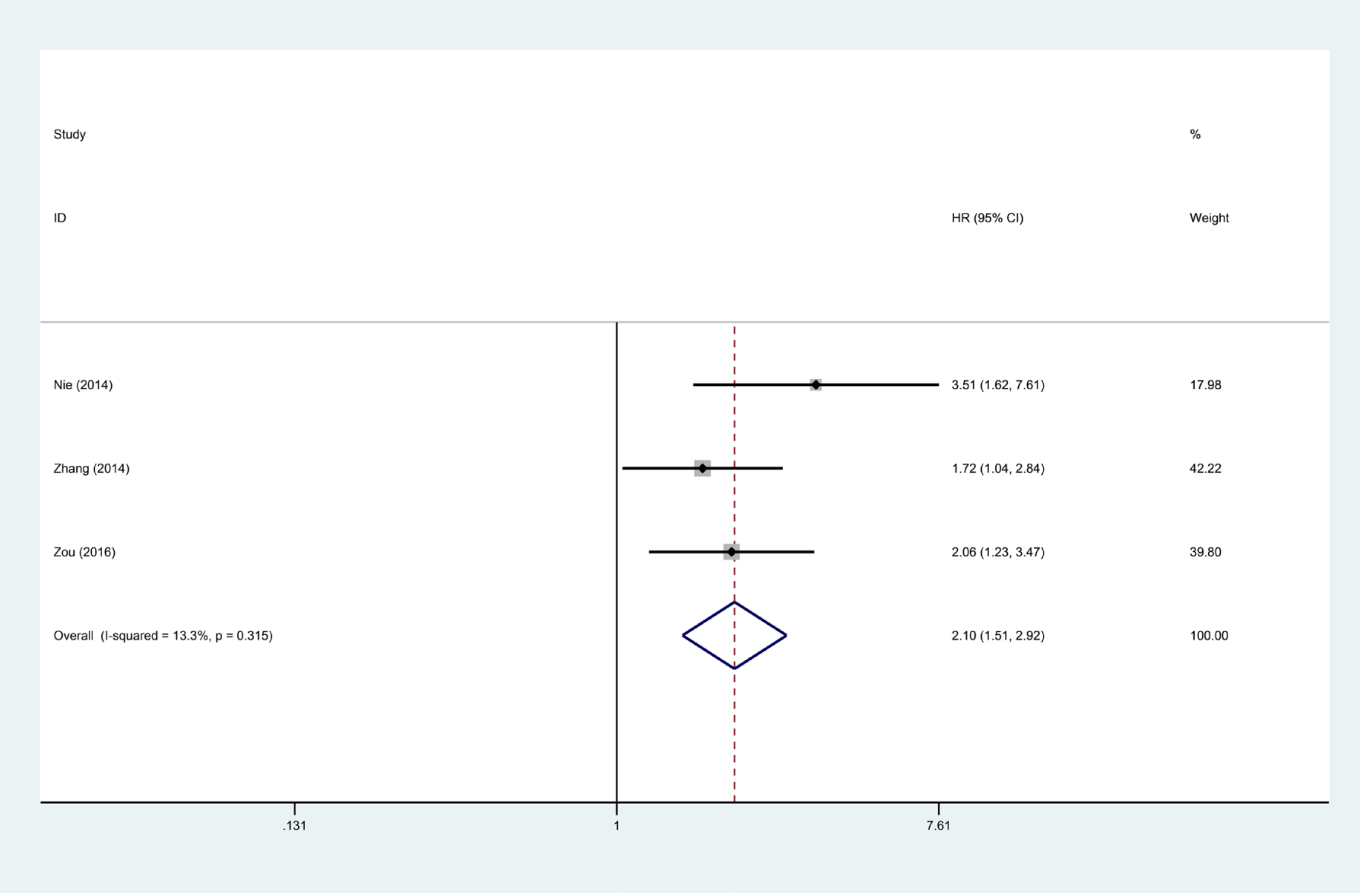
Forest plot for the association between ANRIL expression levels with DFS

Three studies reporting a total of 276 patients were selected for analysis of the association between the lncRNA ANRIL and DFS. The fixed-effects model was adopted as a result of the low heterogeneity (I^2^ = 13.3%, *P* = 0.315). Analysis showed that ANRIL expression was significantly associated with DFS (HR = 2.10, 95% CI: 1.51–2.92, *P* < 0.001). These results demonstrate that higher expression of ANRIL is positively correlated with poor DFS in human cancer (Figure 3).

### Association between lncRNA ANRIL and OS

**Figure 4:**
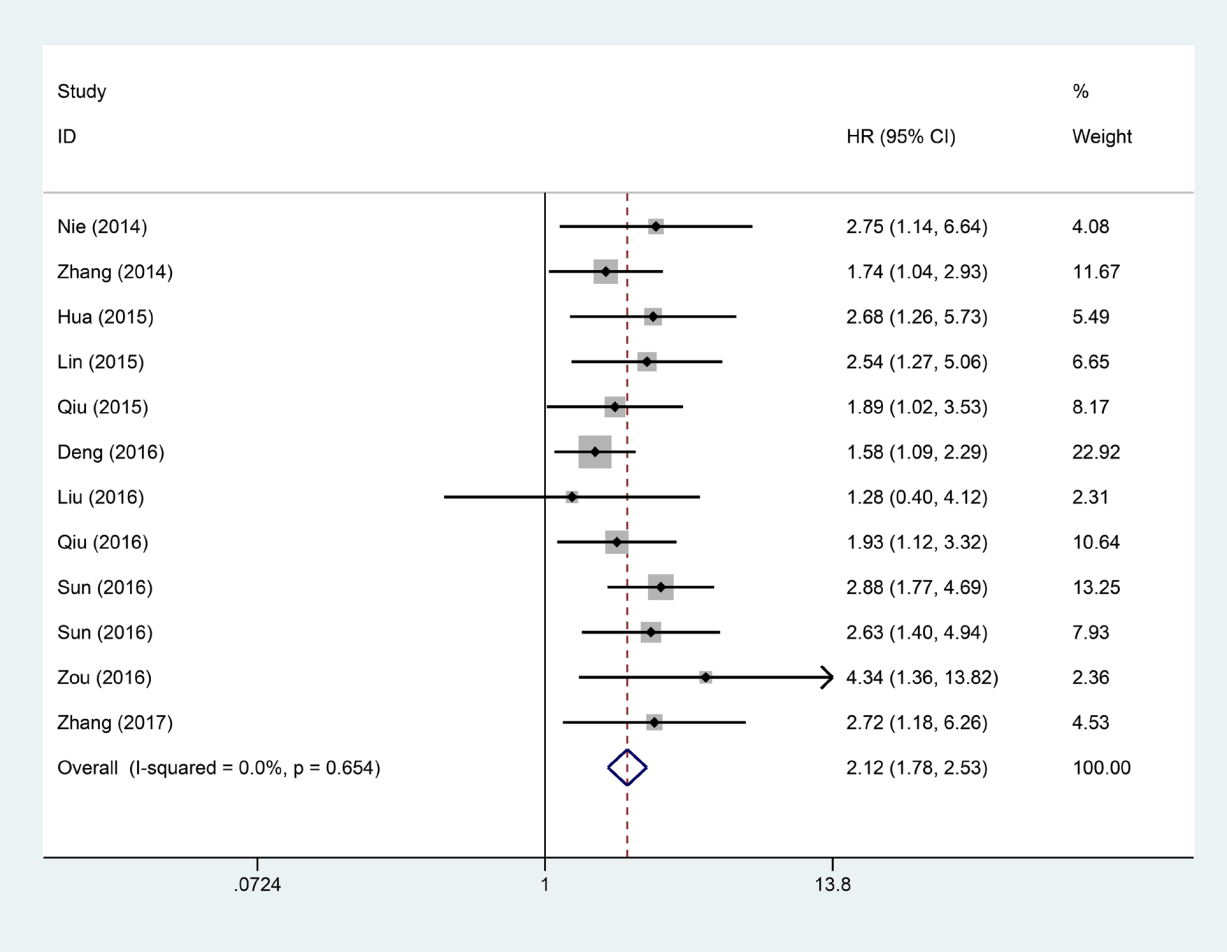
Forest plot of the pooled HRs of elevated lncRNA ANRIL expression for OS in different cancer types

To evaluate the association between the expression level of ANRIL and OS in all patients with cancer, data for pooled HRs and 95% CI of overall survival were collected from the 12 studies with a total of 1067 patients. Analysis showed that there was a significant association between high ANRIL expression and poor OS in patients with cancer (pooled HR = 2.12, 95% CI: 1.78–2.53, *P* < 0.0001) by a fixed-effects model (I^2^ = 0%, *P* = 0.654) (Figure 4).

Although there was no significant between-studies heterogeneity, subgroup analysis via fixed effect model was performed on cancer type, sample size, analysis type, and follow-up time (Table 4). Analysis showed that high expression of ANRIL was significantly associated with shorter OS in patients with cancers of the digestive system (HR = 2.02, 95% CI: 1.62–2.52, *P* < 0.00001) and non-digestive systems (HR = 2.31, 95% CI: 1.72–3.10, *P* < 0.00001). A significant association was also found between high ANRIL expression and OS of patients in articles with sample sizes both equal to or less than 100 (HR = 2.17 95% CI: 1.75–2.68, *P* < 0.00001) and greater than 100 (HR = 2.01, 95% CI: 1.46–2.78, *P* < 0.00001). Furthermore, a strong relationship was shown between ANRIL and OS of patients, as indicated by both multivariate analysis (HR: 2.17, 95% CI: 1.68–2.79, *P* < 0.00001) and non-multivariate analysis (HR: 2.08, 95% CI: 1.62–2.67, *P* < 0.00001). In addition, the high expression level of ANRIL was significantly associated with poor OS of patients with cancer, both equal to or greater than 5 years (HR: 2.07, 95% CI: 1.71–2.49, *P* < 0.00001) and less than 5 years (HR: 2.71, 95% CI: 1.53–4.82, *P* < 0.00001) (Table 4).

**Table 4:** Results of subgroup analysis of pooled HRs of OS of patients with overexpression of lncRNA ANRIL

Stratified analysis	No. of studies	No. of patients	HR/OR (95%CI)	*P*-value	Heterogeneity		
					I^2^ (%)	*P*-value	Model
Cancer type							
non-digestive system	6	466	2.31 (1.72, 3.10)	< 0.00001	0.0	0.803	Fixed effects
digestive system	6	601	2.02 (1.62, 2.52)	< 0.00001	0.0	0.325	Fixed effects
Sample size							
≤ 100	9	768	2.17 (1.75, 2.68)	< 0.00001	0.0	0.484	Fixed effects
> 100	3	299	2.01 (1.46, 2.78)	< 0.00001	0.0	0.604	Fixed effects
Analysis type							
Non-multivariate	5	457	2.08 (1.62, 2.67)	< 0.00001	25.9	0.249	Fixed effects
Multivariate	7	610	2.17 (1.68, 2.79)	< 0.00001	0.0	0.784	Fixed effects
Follow-up time							
< 5	2	160	2.71 (1.53, 4.82)	< 0.00001	0.0	0.965	Fixed effects
≥ 5	10	907	2.07 (1.71, 2.49)	< 0.00001	0.0	0.654	Fixed effects

### Publication bias

**Figure 5:**
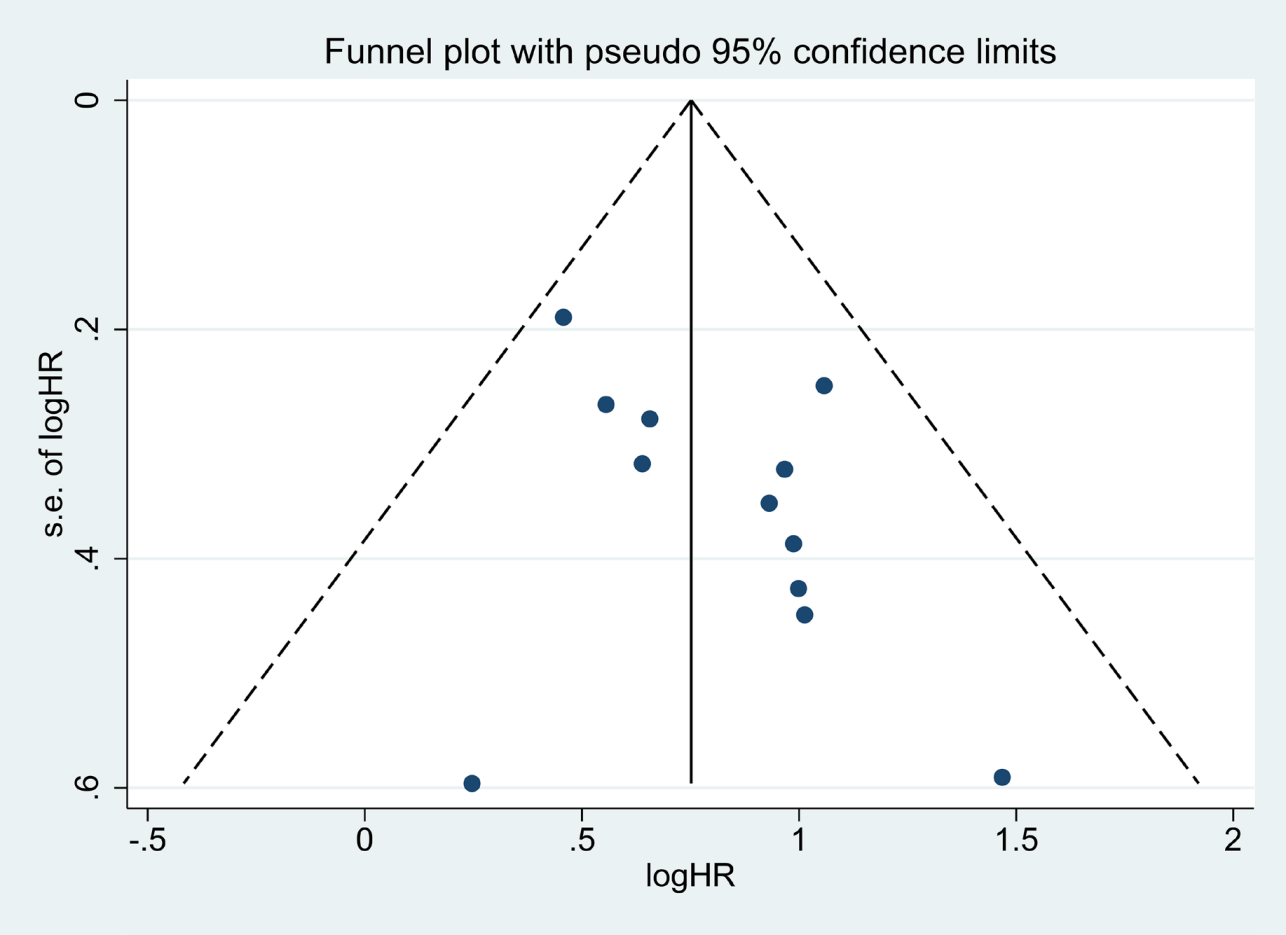
Funnel plot of the publication bias for the analysis of the independent role of ANRIL in OS in the different cancer types

The funnel plot and Egger's test were performed to assess the publication bias of the present meta-analysis. The shape of the funnel plot was roughly symmetrical without obvious evidence of asymmetry (Figure 5). Because of the small sample size of LNM, TNM, DFS, and other groups, publication bias analysis was not conducted.

### Sensitivity analysis

**Figure 6:**
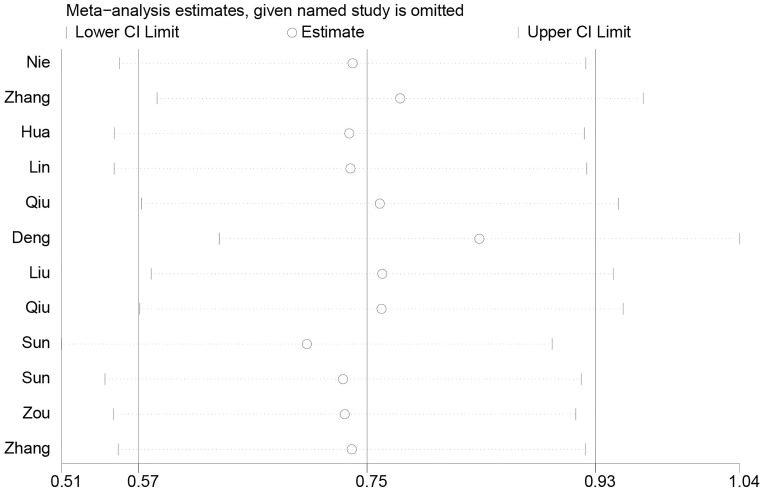
Sensitivity analysis of the effect of individual studies on the pooled ANRIL and OS of patients

To evaluate the impact of a single study on the overall meta-analysis, sensitivity analysis was performed by omitting each eligible study at a time among the total population. When each study was sequentially excluded, the results of the analysis were not significantly affected (Figure 6).

## DISCUSSION

The lncRNA ANRIL is up-regulated in a variety of malignant tumors, acting as a novel oncogene in tumorigenesis and progression, promoting tumor cell proliferation, apoptosis, and metastasis, and reducing survival [19, 22, 23, 29, 30]. Although the role of ANRIL in therioma is not yet clear, a number of studies have addressed this issue. Sun et al. [29] found that high expression of ANRIL was significantly associated with TNM staging, lymphatic metastasis and poor prognosis in colon cancer. Cell mobility and invasiveness were clearly decreased by down-regulating ANRIL expression. Studies of the association between ANRIL and lymphatic metastasis revealed that ANRIL acts as a driver for lymphangiogenesis via the upregulation of LYVE-1, VEFG-C, and VEGFR-3. Yap et al. [32] reported that the lncRNA ANRIL directly colligates polycomb group proteins to INK4a and INK4b loci, reducing the expression of p16INK4a, p14ARF, and p15INK4b. In particular, p16INK4a and p15INK4b are well-known inhibitors of the cyclin-dependent kinase 4 and p14ARF; furthermore, the interaction between p14ARF and murine double minutesute 1 strongly enhances the stability of p53. Accordingly, ANRIL expedites the cell cycle in tumor cells by suppressing or activating p16INK4a, p14ARF, and p15INK4b [29]. Additionally, the inhibition of cervical cancer cell proliferation, migration, and invasion following ANRIL inhibition may occur via the PI3K/Akt pathway [24].

LncRNA ANRIL acts as an oncogene and plays a significant role in GC progression, involving the epigenetic suppression of miR-99a/miR-449a in Trans by binding to PRC2, thus establishing a positive feedback loop to expedite GC cell proliferation [25]. ANRIL promotes the invasion and metastasis of tumor cells by down-regulating the expression of p15INK4B through suppression of the TGF-β/Smad signaling pathway in thyroid cancer [27]. Additionally, ANRIL induces tumor cell proliferation by up-regulating the expression of Bcl-2 and down-regulating P15INK4B expression in epithelial ovarian cancer [28].

The present meta-analysis demonstrated that overexpression of ANRIL is correlated with poor clinical outcomes in patients with cancer. We investigated the association between the expression of ANRIL and clinicopathological features; our meta-analysis showed that overexpression of ANRIL is strongly associated with LNM (OR = 4.77, 95% CI: 2.30–9.91, *P* < 0.001) and clinical stage (OR = 3.44, 95% CI: 1.68–7.08, *P* < 0.001), whereas there was no significant association between high ANRIL expression and histological grade (OR = 1.42, 95% CI: 0.32–6.27, *P* = 0.646) or tumor size (OR = 1.77, 95% CI: 0.63–4.93, *P* = 0.278). However, there was extensive heterogeneity between ANRIL expression level, LNM, and clinical stage. As the heterogeneity may affect the results of meta-analysis, we performed a sensitivity analysis, demonstrating that the existing heterogeneity significantly decreased for LNM. Analysis showed that the heterogeneity was significantly diminished after the Zhang 2014 study was excluded (I^2^ = 43, *P* = 0.119) without affecting the results (OR = 6.03, 95%CI: 3.94–9.25, *P* < 0.0001). Subsequent clinical stage analysis showed that heterogeneity was significantly diminished after the Sun 2016 study was excluded (I^2^ = 0%, *P* = 0.574) also without altering the results (OR = 4.61, 95% CI: 3.22–6.61, *P* < 0.0001) (Supplementary Figure 2). Therefore, the Zhang 2014 and Sun 2016 studies significantly affected heterogeneity of LNM and clinical stage, respectively. Further analysis indicated that heterogeneity may be caused by the following: (1) sample size and sample collection procedures in the study; (2) different reaction conditions or reaction system procedures of RT-qPCR; or (3) difference in cut-off value.

Moreover, when the relationship between the lncRNA ANRIL and DFS was explored, the analysis showed that higher expression of ANRIL was positively correlated with poor DFS (HR = 2.10, 95% CI: 1.51–2.92, *P* < 0.001). We next analyzed the relationship between high ANRIL and OS, demonstrating that ANRIL expression is correlated with shorter OS (HR = 2.12, 95% CI: 1.78–2.53, *P* < 0.0001). Subgroup analysis indicated a significant relationship between ANRIL and OS in cancers of the digestive system (HR = 2.02, 95% CI = 1.62–2.52, *P* < 0.00001) and non-digestive systems (HR = 2.31, 95% CI: 1.72–3.10, *P* < 0.00001); significant association was also found between ANRIL and OS in articles with sample sizes both equal to or less than 100 (HR = 2.17 95% CI: 1.75–2.68, *P* < 0.00001) and greater than 100 (HR = 2.01, 95% CI: 1.46–2.78, *P* < 0.00001). Furthermore, a strong relationship was shown between ANRIL and OS in articles reporting multivariate analysis (HR: 2.17, 95% CI: 1.68–2.79, *P* < 0.00001) and non-multivariate analysis (HR: 2.08, 95% CI: 1.62–2.67, *P* < 0.00001). The expression level of ANRIL was significantly associated with the follow-up times of OS of patients with cancer, both equal to or greater than 5 years (HR: 2.07, 95% CI: 1.71–2.49, *P* < 0.00001) and less than 5 years (HR: 2.71, 95% CI: 1.53–4.82, *P* < 0.00001).Taken together, the results show that high expression of ANRIL serves as a potential predictive factor for lymph node metastasis and poor prognosis in various human cancers.

Nevertheless, this meta-analysis has several limitations. First, only 13 studies were included in this analysis, most of which had a small sample size. Second, the scope of our research results may be limited as all the included studies were from China and, consequently, our results might give rise to potential ethnic bias and only be applicable in this ethnic group. Third, the research results may be affected by the estimation of HR and 95% CIs from Kaplan-Meier curves in some articles. Fourth, many articles reported positive results; this may have occurred because negative results are much more difficult to publish. Finally, this study is a retrospective analysis. All the articles included are retrospective articles, which indicated that the included studies suffered from a moderate risk of bias.

In conclusion, overexpression of the lncRNA ANRIL is significantly related to LNM, TNM stage, and poor prognosis. Our results showed that lncRNA ANRIL may serve as a novel biomarker of lymph node metastasis and prognosis in human cancer. However, larger sample sizes and studies of other ethnic groups are required to affirm the prognostic significance of this lncRNA in human cancers.

## MATERIALS AND METHODS

### Literature retrieval to identify relevant studies for meta-analysis

In accordance with standard meta-analysis guidelines, two authors (Han Wang and Yang Liu) independently searched relevant articles in PubMed, Web of Science, Medline, OVID, and Embase, using “ANRIL,” “LncRNA ANRIL,” “long noncoding RNA ANRIL,” and “antisense noncoding RNA in the INK4 locus” as keywords. The literature search was performed to include articles published up to July 14, 2017. The reference lists of retrieved articles were also searched manually to ensure the inclusion of eligible studies.

### Selection criteria for study inclusion

Inclusion criteria were as follows: (1) articles exploring the association between ANRIL expression and cancer prognosis; (2) patients with cancer were divided into high and low groups according to the expression levels of ANRIL, which was defined by each study; (3) articles describing related clinicopathologic parameters, such as lymph node metastasis, TNM stage, histologic grade, and tumor size; (4) the inclusion of sufficient data for the computation of odds ratios (OR) and corresponding 95% confidence intervals (CI). Exclusion criteria were as the follows: (1) duplicate publications; (2) reviews, letters, case reports, non-human research; and (3) articles without usable data.

### Data extraction from relevant studies

Data extractions were independently performed by three authors (WH, LY, and ZJH) according to inclusion and exclusion criteria. Disagreements were resolved by discussion among authors (ZYT and YG). The following information was recorded: (1) first author, (2) publication date, (3) country of origin, (4) cancer type, (5) sample size, (6) tumor stage, (7) ANRIL expression detection method, (8) cut-off value, (9) number of groups with high ANRIL and low ANRIL expression, (10) HRs, and corresponding 95% CIs for OS and DFS, and (11) follow-up period. Moreover, clinicopathologic parameters (lymph node metastasis, TNM stage, histologic grade and tumor size) were recorded from all retrieved articles. The survival data were directly applied if an article stated the detailed HRs and 95% CIs for survival; otherwise, the Engauge Digitizer 4.1 (http://digitizer.sourceforge.net/) was used to extracted survival data from Kaplan-Meier curves.

### Statistical analysis

The study quality was assessed according to the Newcastle-Ottawa Scale (NOS). All statistical analyses were conducted using Stata statistical software version 12.0 (Stata Corporation, College Station, Texas, USA). Chi square-based *Q* test and I^2^ statistics were performed to measure the heterogeneity of the eligible studies. The fixed effects model was adopted in the absence of heterogeneity (*P* > 0.1 and I^2^ < 50%), and the random-effects model was applied if heterogeneity was determined (*P* < 0.1 and I^2^ > 50%). Egger's test was applied to evaluate the potential publication bias, and the stability of the results was assessed through sensitivity analysis. *P* < 0.05 was deemed to represent statistical significance.

## SUPPLEMENTARY MATERIALS FIGURES


