# Supplementary material for: Long noncoding RNA ANRIL as a novel biomarker of lymph node metastasis and prognosis in human cancer: a meta-analysis
Source: Oncotarget. 2017 Oct 11;9(18):14608–18. doi: 10.18632/oncotarget.21825 (PMC5865693; doi:10.18632/oncotarget.21825)
Supplement: Supplementary file 1 [file oncotarget-09-14608-s001.pdf]

# Long noncoding RNA ANRIL as a novel biomarker of lymph node metastasis and prognosis in human cancer: a meta-analysis

## SUPPLEMENTARY MATERIALS

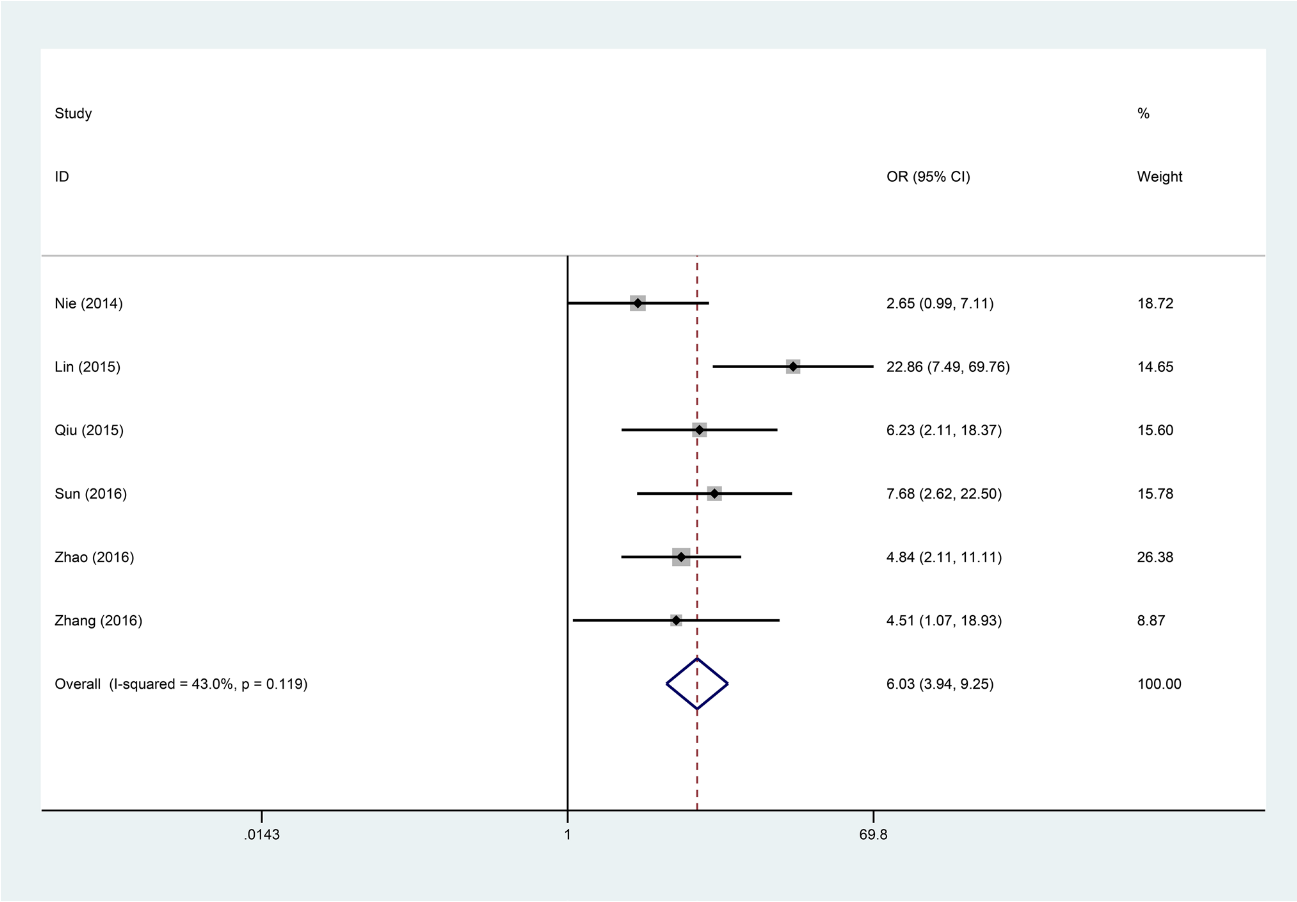


**Supplementary Figure 1: Forest plot for the association between ANRIL expression levels with LNM after the Zhang 2014 study was excluded.**

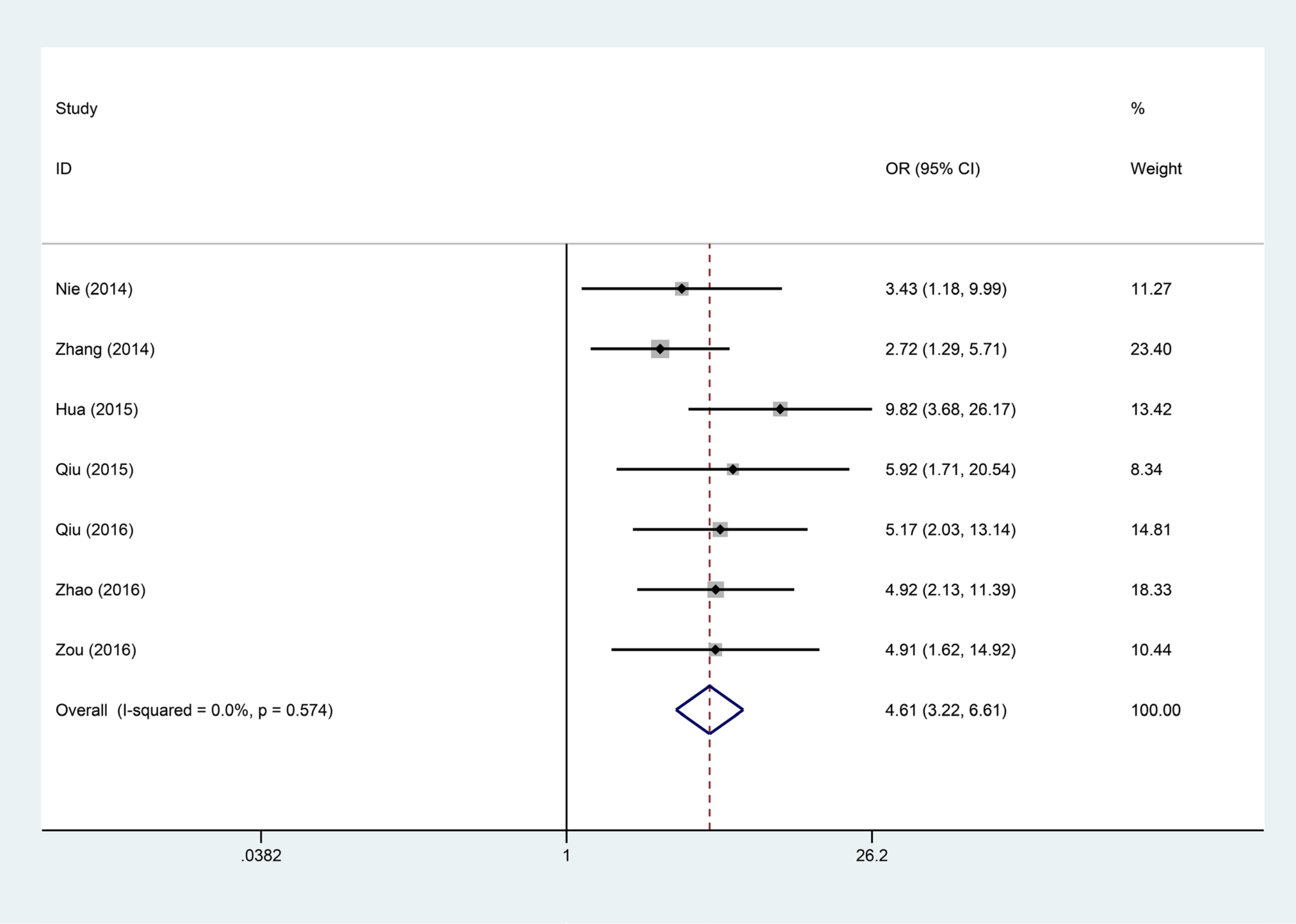


**Supplementary Figure 2: Forest plot for the association between ANRIL expression levels with TNM after the Sun 2016 study was excluded.**
